# Decreasing incidence of registered hydatidiform moles in Denmark 1999–2014

**DOI:** 10.1038/s41598-020-73921-4

**Published:** 2020-10-12

**Authors:** Helle Lund, Mogens Vyberg, Helle Højmark Eriksen, Anni Grove, Annette Østergaard Jensen, Lone Sunde

**Affiliations:** 1 Department of Pathology, Aalborg University Hospital, Ladegaardsgade 3, 9000 Aalborg, Denmark; 2 Department of Clinical Medicine, Aalborg University, Søndre Skovvej 15, 9000 Aalborg, Denmark; 3 Unit of Epidemiology and Biostatistics, Aalborg University Hospital, Søndre Skovvej 15, 9000 Aalborg, Denmark; 4 Department of Biomedicine, Aarhus University, C. F. Møllers Allé 6, 8000 Aarhus C, Denmark; 5 Department of Clinical Genetics, Aalborg University Hospital, Ladegaardsgade 5, 9000 Aalborg, Denmark

**Keywords:** Diseases, Medical research

## Abstract

Incidences of hydatidiform mole (HM) registered in European countries varies from 0.98/1000 to 2.17/1000 deliveries, while higher incidences have been reported in other parts of the world. We calculated the incidence by selecting data on HMs classified as ”first”, “second” and “third” from 01.01.1999 to 31.12.2014 registered in the Danish Pathology Registry, which we previously showed to be the most complete data source on the number of HMs in Denmark. In the study period, 1976 first HMs were registered; 1080 (55%) were classified as PHMs (partial HMs) and 896 (45%) as NPHMs (HMs not registered as PHMs). The average incidence of HM was 1.98/1000 deliveries. The incidence of PHM was 1.08/1000 deliveries and the incidence of NPHM was 0.90/1000 deliveries. Forty HMs were registered as second HMs; 85% (34/40) were of the same histopathological type as the first HM. The registered incidence of HM decreased from 2.55/1000 deliveries in 1999 to 1.61/1000 deliveries in 2014 (p < 0.005). The decrease in the incidence of HM was identical with a decrease in the incidence of PHM. New medical practices such as medical abortion and only forwarding selected pregnancy products for histopathologic examination may cause a declining number of HMs registered.

## Introduction

Hydatidiform mole (HM) is an abnormal human pregnancy and the most common gestational trophoblastic disease. Based on morphological criteria, HMs can be classified as complete hydatidiform moles (CHM) or partial hydatidiform moles (PHMs)^1^. HM is a rare cause of miscarriage, but a frequent cause of gestational trophoblastic neoplasia^2^. HMs have been recognized for more than 1000 years^3^. In Europe, the reported incidence of HM varies from 0.98 to 2.17 per 1000 deliveries in the period 1997–2013, while higher incidences have been reported from other parts of the world^4–9^.

Several factors limit the accuracy of the incidences estimated. First and foremost, we know little about very early pregnancies. Some molar conceptuses may vanish before the pregnancy is recognized. And similarly, we do not know the correct denominator, as the total number of pregnancies in a population can only be estimated with uncertainty. Therefore various “surrogate denominators” are being used, such as “number of viable pregnancies” and “number of deliveries”. In addition many reports concern the observations in a hospital, a referral center, or similar, introducing a risk of referral bias^9–11^. Furthermore, the accuracy of the incidences estimated will be influenced by other factors, such as the ability to uniquely identify each individual in a population and the completeness of the registries used.

In Denmark, each individual is unambiguously identifiable due to the civil personal registration (CPR) number, assigned at birth or immigration. Systematic registration of births in Denmark started in 1968^12^, and since 2006 the number of induced abortions and miscarriages has been registered in the Danish Database for Early Pregnancy and Abortion (TiGrAb, se description below). Further, women with a HM are registered in three governmental registries: the Danish National Patient Registry^13^, the Danish Cancer Registry^14^, and the Danish Pathology Registry (DPR)^15^. We recently proved the DPR to be the most complete data source for research on the number of HMs in Denmark^16^. Here we present the incidence of HM in Denmark for the period 1999–2014, estimated from data in the DPR. We also review the incidences estimated in other population-based studies published in the period 01.01.1990–31.12.2019. The average incidence of registered HMs in Denmark was among the highest reported incidences in Europe. Further, we observed a significantly decreasing incidence of PHM over time.

## Methods

We conducted a population-based study in Denmark (approximately 5.5 million inhabitants). The entire population receive tax-supported health care from the Danish National Health Service without out-of-the-pocket expenses for hospital care. Since 1968, the Danish Civil Registration System has kept up-to-date electronic records on the date of birth, gender and changes in vital status for all Danish residents. Each resident in Denmark is assigned a unique 10-digit CPR number, which allows unambiguous linkage among Danish registries^17^.

### The Danish Pathology Registry

The DPR holds nationwide data on pathology investigations and is managed by the Danish National Board of Health. The DPR was established in 1997, however computer-based recording of pathological specimens was initiated in Denmark in the 1970s. Since 1999, reporting to the DPR automatically takes place through the Danish Pathology Data Bank, a nationwide database working as a routine online tool for all pathology departments in Denmark^18^. For each specimen, data on the patient, date of registration, gross and microscopy descriptions, and diagnoses are registered. Coding is performed using the Danish modification of the Systemized Nomenclature of Medicine (SNOMED, https://www.patobank.dk/index.php?ID=4&lang=da). The data quality is ensured by the approval of all diagnostic statements in the pathology report by a pathologist, and a debugging system that ensures that all reports are given at least one code for topography and one code for morphology. The registration in the DPR is performed on-line as an automated function linked to the electronic sign out of a pathology report.

### Identification of hydatidiform moles

In the DPR, data are registered for each specimen analyzed. Initially we retrieved data on all specimens registered with a SNOMED morphology code for HM in the DPR from 01.01.1987 to 31.12.2014. In this data set, each specimen was listed with the CPR number of the woman and the date of registration in the DPR, along with a number of other variables. We grouped the specimens according to the CPR number. In each group, we identified the woman’s first HM by the CPR number and the first date of registration of a specimen. Further, in each group, all specimens registered 0–180 days after the first date of registration were ignored, whereas a specimen with the earliest date of registration later than 180 days after the first date of registration was identified as a second HM. A third HM was identified by the CPR number and the earliest date of registration more than 180 days after the date of registration of a second HM, and so on.

To estimate the incidence of HM in the period 01.01.1999–31.12.2014, we selected data on HMs classified as “first HMs” with a date of registration in this period. To calculate the frequency of subsequent HMs in the period 01.01.1999–31.12.2014, we selected data on HMs classified as “second HMs” or “third HMs” in the period. No HMs were classified as “fourth HMs”.

Throughout the period 01.01.1987–31.12.2014, the SNOMED morphology code M91030 has been used for “partial hydatidiform mole” (Table 1). The code M91000 was used for “hydatidiform mole” until 31.12.2013 and for “complete hydatidiform mole” thereafter. The code M910A0 for “hydatidiform mole, not otherwise specified” was introduced in 01.01.2014. The code ÆYYY00 is a moderator code indicating “suspicion of”. The moderator code can be used as a supplement to the morphology codes.

**Table 1:** SNOMED codes for hydatidiform mole in the Danish Pathology Registry 1999–2014.

Code	Period	Diagnosis
M91000	01.01.1999–31.12.2013	Hydatidiform mole
M91000	01.01.2014–31.12.2014	Complete hydatidiform mole
M910A0	01.01.2014–31.12.2014	Hydatidiform mole, not otherwise specified
M91030	01.01.1999–31.12.2014	Partial hydatidiform mole

We defined two groups of HMs: HMs encoded M91030 were defined as PHMs (partial HMs), and HMs encoded M91000 or M910A0 were defined as NPHM (HMs, not registered as PHMs).

### The Danish Medical Birth Registry

We retrieved the total number of deliveries from the Danish Medical Birth Registry (MBR), which was established in 1968 and computerized in 1973^12^. The MBR is a national health registry. In 1997, electronic registration of births from the Danish National Patient Registry^13^ replaced the previously used paper forms^19^. The registry contains information on both live births and stillbirths. Before 01.01.2005, a pregnancy which concluded at a gestational age of 28 weeks or later, was considered as resulting in a birth. From 01.01.2005 a pregnancy, which concluded at a gestational age of 22 weeks or later, was considered as resulting in a birth.

### The Danish Database for Early Pregnancy and Abortion

We retrieved the number of induced abortions, miscarriages, ectopic pregnancies, and pregnancies of unknown localization from the database TiGrAb (The Danish Database for Early Pregnancy and Abortion; https://www.sundhed.dk/content/cms/67/4667_%C3%A5rsrapport-2016_endelig_anonymiseret.pdf). The database is a clinical quality database managed by the National Board of Health^20^. TiGrAb was established in 2006 and the content is based on data from the Danish National Patient Registry^13^.

### Statistical analyses

The incidence of HM per delivery was calculated as the number of first HMs registered in the DPR in the period 01.01.1999–31.12.2014 divided by the total number of deliveries registered in the MBR in the same period. To evaluate the association between the incidence of HM and age, we stratified the data according to the maternal age at the date of registration of the specimen. For the period 01.01.2007–31.12.2014, the number of pregnancies was available. We calculated the incidence per pregnancy by dividing the number of first HMs registered in the DPR in the period 01.01.2007–31.12.2014 by the total number of pregnancies ending in the same period. The total number of pregnancies was calculated by adding the number of deliveries (retrieved from the MBR) and the number of HMs (identified in the DPR) to the number of induced abortions, miscarriages, ectopic pregnancies, and pregnancies of unknown localization retrieved from TiGrAb (Supplementary material, part A). Poisson regression model (Wald test) was used to compare incidence rates. Statistical analyses were performed with STATA software (StataCorp. 2013. Stata Statistical Software: Release 13. College Station, TX: StataCorp LP). The study was registered at the Danish Data Protection Agency (record no 2014-41-3541).

### Literature search

To identify incidences of HM reported from population-based studies, we identified literature in PubMed by searching for “molar pregnanc*” or “hydatidiform” or “gestational trophoblastic disease*” in the title, and “incidence*” in the title or abstract, for papers published in the period 01.01.1999–31.12.2019. The initial search in PubMed resulted in 194 references. By reading the title, abstract, and if necessary the text, the following studies were excluded; review articles, hospital-based studies, institution-based studies, multicenter-studies, and studies from referral centers, as well as studies in non-English language and studies for which details on data source and data management were missing.

## Results

A total of 1976 first HMs were registered in the period 01.01.1999–31.12.2014; 896 (45%) first HMs were classified as NPHMs and 1080 (55%) were classified as PHMs. From 01.01.2014 to 31.12.2014, 34/91 (37%) first HMs were registered as CHMs, 55/91 (60%) were registered as PHMs, and two (2%) were registered as “hydatidiform mole, not otherwise specified” (M910A0). The moderator code indicating “suspicion of” was used in 277 of 1976 first HMs (14%); in 117/896 (13%) of the first HMs classified as NPHMs and in 160/1080 (15%) of the first HMs classified as PHMs (Supplementary material, part B). There was no significant change in the proportion of HMs registered with the moderator code indicating “suspicion of” over time (data not shown).

The mean age of women registered with a first HM classified as a NPHM was 30 years, range 13–56 years; and the mean age of women registered with a first HM classified as a PHM was 30 years, range 14–60 years.

**Figure 1:**
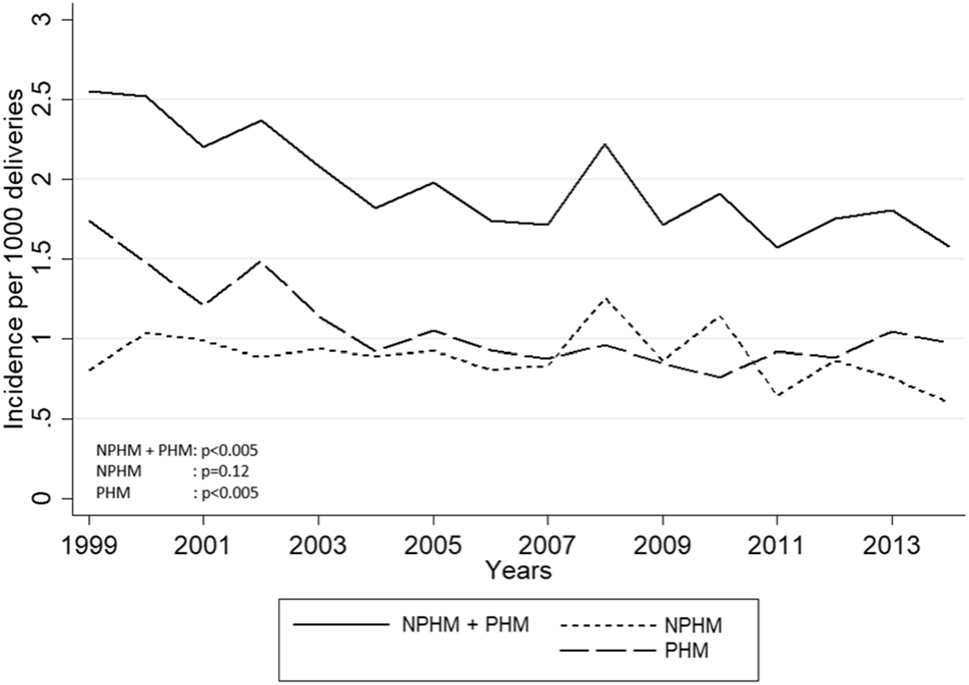
Incidence of hydatidiform mole (HM) in Denmark 1999–2014. *NPHM* HM registered with the code M91000 (hydatidiform mole or complete hydatidiform mole) or M910A0 (hydatidiform mole, not otherwise specified); *PHM* HM registered with the code M91030 (partial hydatidiform mole).

According to the DPR, the average incidence of HM from 1999 to 2014 was 1.98/1000 deliveries (Supplementary material, part B). The incidence of HM classified as NPHM was 0.90/1000 deliveries. The incidence of HM classified as PHM was 1.08/1000 deliveries. By excluding all first HMs with the moderator code, the following minimum average incidences were calculated: HM: 1.70/1000 deliveries, NPHM: 0.78/1000 deliveries, and PHM: 0.92/1000 deliveries. The incidence of HM decreased from 2.55/1000 deliveries in 1999 to 1.61/1000 deliveries in 2014 (p < 0.005). The decrease occurred mainly in the first part of the period (Fig. 1). The decrease in the incidence of HM was almost identical to a decrease in the incidence of PHM. The decrease in the incidence of PHM was also significant (p < 0.005). No significant change over time was observed for NPHM (p = 0.12).

**Figure 2:**
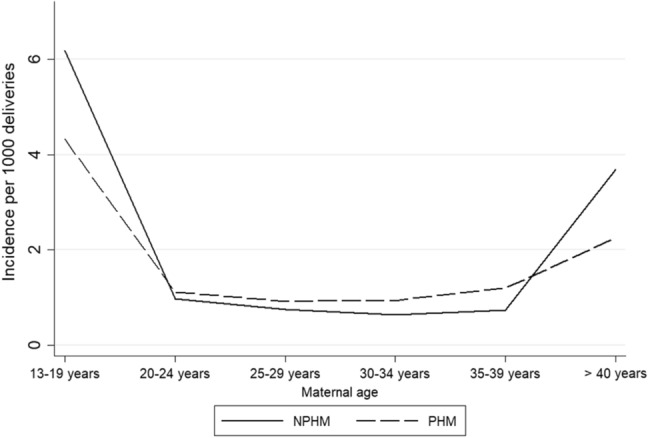
Age specific incidence of hydatidiform mole (HM) in Denmark 1999–2014. *NPHM* HM registered with the code M91000 (hydatidiform mole or complete hydatidiform mole) or M910A0 (hydatidiform mole, not otherwise specified); *PHM* HM registered with the code M91030 (partial hydatidiform mole).

The highest incidence of HM was observed in women younger than 20 years (10.52/1000 deliveries) and in women older than 39 years (5.95/1000 deliveries). The tendency for higher incidences among both young and elderly women was more pronounced for NPHM than for PHM (Fig. 2). However, the absolute number of HM was highest for women between 20 and 39 years (1657/1976, 84%). The proportion of NPHMs in women younger than 20 years and in women older than 39 years was 59% and 62%, respectively, while it was 42% in women 20–39 years old.

In the period 2007–2014, the average incidence of HM was 1.23 per 1000 pregnancies with no obvious change over time (Supplementary material, part C).

In the period 01.01.1999–31.12.2014, 40 second HMs and one third HM were registered. No fourth HM was registered. Thirty-four of the 40 second HMs (85%) were of the same histopathological type as the first HM; 22 second HMs were PHMs and 18 second HMs were NPHMs (Table 2). In 16/16 women registered with a second HM whose first HM was a PHM, the second HM was also a PHM. Twenty-four of 896 (2.7%) women whose first HM was a NPHM, were also registered with a second HM, whereas 16 of 1080 (1.5%) women whose first HM was a PHM, were registered with a second HM. The median interval between the first and second HM was 582 days (Supplementary material, part D). The third HM was a NPHM, and both the first and second HM in this woman were NPHMs. For women having a second HM, the mean age at which the women experienced their first HM was 30 years (range 15–56), and 28 years (range 24–37), respectively, in women where the first HM was a NPHM and a PHM, respectively. The woman with a third HM was 23 years old, when the first HM was registered.

**Table 2:** Frequency and morphological subtype of the second hydatidiform mole (HM) stratified on morphological subtype of the first HM (NPHM or PHM).

First HM	Second HM			Total HMs registered
	NPHM^a^	PHM^b^	Total	
NPHM^a^	18	6	24	896
PHM^b^	0	16	16	1080
Total	18	22	40	1976

*HM* hydatidiform mole, *NPHM* a HM not registered as a PHM, *PHM* partial hydatidiform mole.

^a^HM registered with the code M91000 (hydatidiform mole or complete hydatidiform mole) or M910A0 (hydatidiform mole, not otherwise specified).

^b^HM registered with the code M91030 (partial hydatidiform mole).

Searching the literature for studies reporting the incidences of HM estimated in population-based studies, we identified 17 papers published in 1990–2019 (Table 3). Most studies were from European countries, no studies were from Africa, South America, or Australia.

**Table 3:** Incidence of hydatidiform mole (HM) reported in population-based studies since 1990.

Author (year)	Population	Data source	N/R	Period	No	Incidence per 1000 total (complete HM/partial HM)			
						Pregnancies	Viable conceptions*	Deliveries	Live births
**Scandinavia**									
Present study	Denmark	The Danish Pathology Registry	N	1999–2014	1976			1.98 (0.9^a^/1.08)	
				2007–2014	863	1.23 (0.61^a^/0.62)		1.79	
Olsen et al.^37^	Denmark	The Danish National Patient Registry/The Danish Cancer Registry	N	1977–1992	1520	1.1^b^			
Salehi et al.^26^	Sweden	The Swedish Cancer Register/The Inpatient Register	N	1973–2004	3844			1.2^c^	
Joneborg et al.^5^	Stockholm County, Sweden	The Regional Cancer Register of Stockholm County/The Regional Pathology data base	R	1991–2010	956		1.48^d^	2.08^e^	
Flam et al.^36^	Stockholm County, Sweden	The Regional Cancer Register of Stockholm County/medical records	R	1975–1988	393	0.9^f^		1.5	
Loukovaara et al.^4^	Finland	The hospital discharge registry of the National Research and Development Center for Welfare and Health	N	1975–2001	1659			0.98	
**Europe**									
Savage et al.^34^	England and Wales	Databases of the Trophoblastic Disease Centres at Charing Cross Hospital (London) and Weston Park Hospital (Sheffield)	N	2000–2009	13,583		1.65^g^ (0.70/0.95)	2.13^h^	
Savage et al.^27^	England and Wales	Databases of the Trophoblastic Disease Centres at Charing Cross Hospital (London) and Weston Park Hospital (Sheffield)	N	1998–2007	14,161		1.69^i^	2.17^h^	
Tham et al.^33^	Northern England and North Wales	The Trophoblastic Screening and Treatment Centre, Weston Park Hospital (Sheffield)	R	1991–1999	3637				1.38
Eysbouts et al.^6^	The Netherlands	A national pathology database, PALGA	N	1994–2013	5135			1.36 (0.52/0.67)	
Lybol et al.^11^	The Netherlands	A national pathology database, PALGA	N	1995–2008	3668			1.34 (0.47/0.69)	
Parazzini et al.^25^	Lombardy, Northern Italy	Integrated Patients Database of the Lombardy Region	R	1996–2008	1433	1.04^j^			
**USA/Canada**									
Altman et al.^8^	Nova Scotia, Canada	Nova Scotia Gestational Trophoblastic Disease Registry	N	1990–2005	420	2.20^k^ (0.71/1.45)		2.64^l^ (0.93/1.71)	2.66 (0.94/1.72)
Smith et al.^32^	New Mexico, USA	The New Mexico Tumor Registry	N	1988–1997	389		1.20^m^ (0.81/0.39)		1.42 (0.96/0.46)
**Asia**									
Yuk et al.^22^	South Korea	National Inpatient Sample, extracted records of 13% of patient per year	N	2009–2015	285	1.10^n^ (0.10/0.10)			
Kim et al.^7^	Korea	Korea medical insurance system	N	1991–1994	5855			2.05	
Matsui et al.^23^	Chiba Prefecture, Japan	Questionnaires registration system	R	1985–2002	1876		1. 5^o^ (0.83/0.67)		
Matsui et al.^24^	Chiba Prefecture, Japan	Questionnaires registration system	R	1974–2000	3778				2.27

N/R: N, National studies concerned one country (for USA/Canada: one state/province). R, Regional studies concerned one well-defined region. No: Number of hydatidiform moles.

*Incidences listed here where either referred to as “viable conceptions” and/or it appeared that miscarriages were not included in the denominator.

^a^HM not registered as PHM.

^b^Pregnancies: live births, stillbirths, induced abortions, and spontaneous abortions.

^c^Deliveries: live births and stillbirths from gestational age 28 or later.

^d^Viable conceptions: deliveries and pregnancy terminations.

^e^Deliveries: live births and stillbirths (multiple births counted as one delivery).

^f^Pregnancies: spontaneous, missed, and legal abortions, ectopics and deliveries.

^g^Conceptions: live births, stillbirths, terminations and moles.

^h^Maternities: live births and stillbirths.

^i^Conceptions: live births, stillbirths, terminations, and moles.

^j^Pregnancies: births, spontaneous or induced abortions, ectopic pregnancies and hydatidiform moles.

^k^Pregnancies: live births, stillbirths, induced abortions, miscarriages and ectopic pregnancies.

^l^Deliveries: live births and stillbirths 20 or more weeks of gestational age.

^m^Pregnancies: live births, fetal deaths (stillbirths of > 20 weeks of gestational age or 500 g) and induced abortions (not first trimester abortion or ectopic pregnancies).

^n^Pregnancies: GTD cases, ectopic pregnancies, abortions and deliveries.

^o^Pregnancies: live births, stillbirths and therapeutic abortions.

**Figure 3:**
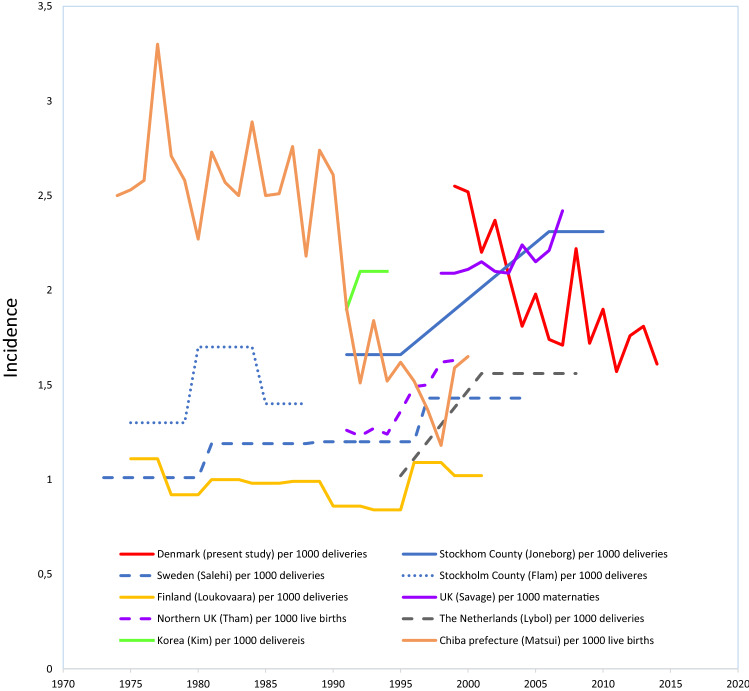
Trends in the incidence of hydatidiform mole (HM) from population-based studies in the period 1990–2019 calculated per 1000 deliveries, maternities or live births.

**Figure 4:**
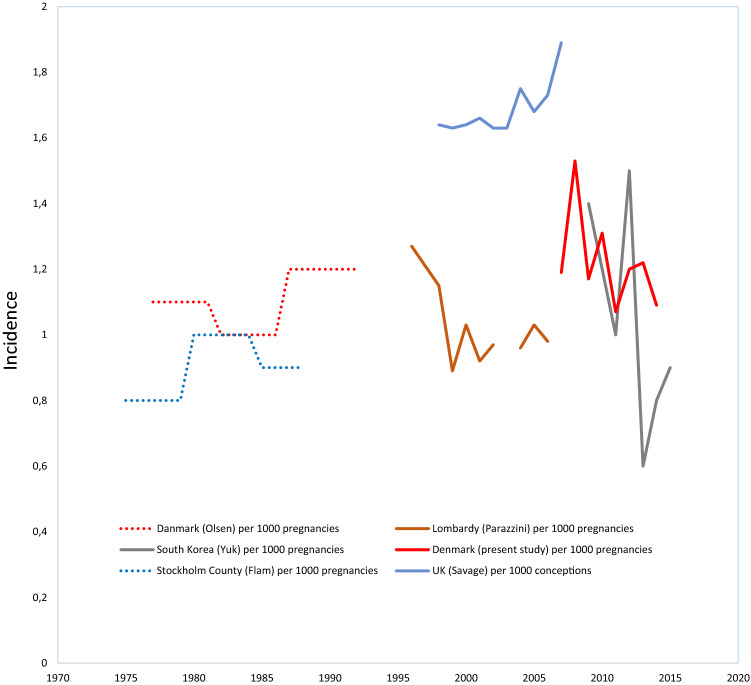
Trends in the incidence of hydatidiform mole (HM) from population-based studies in the period 1990–2019 calculated per 1000 pregnancies or conceptions.

In ten studies the incidence per pregnancy or viable conception was estimated, and in 13 studies the incidence per delivery or live birth was estimated. Furthermore, the four denominators indicated were defined in different ways, or undefined. One example: Among the ten studies reporting incidence per delivery, in five studies delivery was defined in four different ways, and in five studies delivery was not defined (Table 3). Nine studies reported the incidence per delivery/live birth for more than one period and five studies reported the incidence per pregnancy/viable conception for more than one period (Figs. 3 and 4).

## Discussion

We estimated the incidence of HM in Denmark using the DPR, a nation-wide registry with a high completeness, and a high concordance between the description in the pathology report and the SNOMED code^16^. Among the strengths of our study is the population-based design applied in Denmark, where citizens have equal access to health care. Furthermore, unique identification of individuals was possible as CPR numbers are assigned to all Danish citizens. Lastly, to optimize the accuracy of the estimated number of first HMs from 1999 to 2014, we used data for the period 1987–1998 to exclude as many women as possible with a previous HM in that period.

Inherent in a study like this, is the limitation that data on very early spontaneous abortions and medical abortions, are not registered. Also, as the code M91000 was used for “hydatidiform mole” in the period 1999–2013, we were not able to tell which of the HMs encoded M9100 before 2014 were CHMs, and which were HMs that were not specified. However, data from 2014 suggest that most HMs encoded M91000 were CHMs. We did not look into the ethnic origin of the women. However, the number of immigrant women in Denmark is expected to be low^21^. Finally, we cannot exclude that the frequency of subsequent HMs is overestimated as it was not possible to ensure complete gonadotropin remission before diagnosing second or third HMs or underestimated as registration of HMs before 1998 may have been less complete than after.

We calculated the incidence as the number of first HMs registered in the DPR in a given period, divided by the number of deliveries or pregnancies terminated in the same period. Using the total number of specimens given a HM code in the DPR could lead to an overestimation of the incidence of HM, as re-evacuation is performed in some women having had a HM. On the contrary, using the number of women registered in the DPR with a first HM, only, would result in an underestimation of the frequency of HM, as a woman can have more than one HM. To address the latter, we calculated the frequency of subsequent HMs as well.

For the period 1999–2014, we observed a decrease in the incidence of HM registered in Denmark, most pronounced for the first part of the period. Several studies have reported decreasing incidences of HM in Asia^22–24^. In contrast, since 1990 this has only been reported once from Europe: In the Lombardy region of Italy the registered incidence of HM per pregnancy decreased from 1996 to 2008, tentatively explained by the authors by a decreasing incidence among Asian women residing in the region^25^.

Most European studies describe an increase in the incidence of HM per delivery over time (Fig. 3) and in most papers it is speculated, that the increasing incidence could be due to an increased use of ancillary diagnostic techniques^5,6,11,26,27^. Immunohistochemical staining of p57, in situ hybridization, flow cytometry, and genotyping are useful ancillary techniques in the diagnostics of HMs and may lead to an increased number of conceptuses diagnosed as HMs. However, the increased use of ancillary techniques may also result in a decrease in the observed incidence, as some suspected HMs can be ruled out as moles. We did not observe any change over time in the proportion of HMs registered with the moderator code indicating “suspicion of”, suggesting that the frequency of conceptuses with a mole(-like) phenotype that is not diagnosed with certainty, has not changed significantly during the period.

The decreasing incidence of registered HMs in Denmark could possibly be explained by the gynecologists sending fewer products of conception for histopathological examination due to the increased use of medical abortions for both induced and missed abortions^28^. In early pregnancy failures, histological examination remains the gold standard for diagnosing HMs, as routine pre-evacuation ultrasound examination identifies less than 50% of HMs, the majority sonographically appearing as missed abortion or incomplete miscarriage^29^. From 2001 to 2016 the fraction of Danish women with spontaneous abortion having evacuation decreased from 22.8% to 5.9%. Likewise, the fraction of women with missed abortion having evacuation decreased from 71.7% to 42.5%^30^. Although the risk of persistent trophoblastic disease is low following a PHM^2^, it is very important that all products of conception with a suspicion of a HM, are sent for histopathological examination.

Due to the risk of referral bias in hospital-based studies, incidences are preferably estimated in population-based studies^9–11^. These can be either regional, concerning a well-defined region, or national, concerning an entire country, state, or province. However, even from population-based studies, quite varying incidences have been reported. In a nationwide study from Nova Scotia, an average incidence of 2.64/1000 deliveries in 1990–2005 was observed (Table 3). No information about time trend was given^8^. Also, two region-based studies from Asia reported higher average incidences than the Danish incidence: 2.27/1000 live births in Japan (1974–2000) and 2.05/1000 deliveries in Korea (1991–1994)^7,24^. This may be an indication that ethnicity is a risk factor for molar pregnancies^31–33^. However, as our study period (1999–2014) included years that were later than the study period in these studies, the discrepancies may be also, partially, be explained by a tendency for a declining incidence of registered HMs over time.

In a region-based study, Joneborg et al. found an average incidence of 2.08/1000 deliveries in Stockholm County, Sweden for the period 1991–2010, by using all available registries, including a pathology database^5^. However, the incidence observed may be influenced by the fact that the region studied included the capital of Sweden: a higher average maternal age and a higher proportion of women of Asian descent could cause a higher incidence in Stockholm than in other parts of Sweden^5^. Savage et al. estimated the average incidence in England and Wales to 2.17/1000 maternities for the period 1998–2007^27^ and 2.13/1000 maternities for the period 2000–2009^34^. The average incidence of registered first HMs in Denmark in the period 2000–2009 was 2.04/1000 deliveries, making the difference between the British and the Danish incidences less obvious.

Apart from the observations in Stockholm county and UK, the average Danish incidence was higher than the incidences observed in all other European population-based studies published after 1990 (Table 3): using data from clinical registries, average incidences for Sweden, Finland, and Italy of 0.98–1.5/1000 deliveries^4,26,35^ and 0.9–1.04/1000 pregnancies have been reported for various periods from 1973 to 2008^25,36^. These incidences are even lower than the minimum average incidence in our study estimated after excluding cases with the moderator code indicating “suspicion of”. This may partly be explained by the fact that the completeness of clinical registries tend to be lower than the completeness of pathology registries^16^. However, even two studies from the Netherlands, using data from a national pathology registry, found lower average incidences than we did: 1.34/1000 deliveries from 1995 to 2008^11^, and 1.36/1000 deliveries from 1994 to 2013^6^. Thus, although it is possible that the DPR is more complete than the registries in many other European countries, we cannot exclude that the incidence of HM is higher in Denmark than in other parts of Europe.

Olsen et al.^37^ reported an average incidence of HM in Denmark of 1.1/1000 pregnancies in 1977–1992 based on data from the Danish National Patient Registry and the Danish Cancer Registry, whereas we observed an average incidence of 1.23 HM per 1000 pregnancies in 2007–2014. The main explanation for the higher incidence observed in our study may be a higher completeness of the DPR compared to the clinical registries used by Olsen^16,35^. Assuming a completeness of 0.73 of the Danish National Patient Registry relative to the DPR^16^, Olsen et al. would have estimated the incidence for the period 1977–1992 to be 1.5/1000 pregnancies if they had used the DPR. However, we do not know the completeness of the two registries in the period 1977–1992. Furthermore, Olsen et al. used data from both the Danish National Patient Registry and the Danish Cancer Registry. Thus, the incidence estimated for the two periods should be compared with caution. Other population-based studies reported quite varying incidences per pregnancy. Studies from Sweden, Italy, UK, US, South Korea and Japan found average incidences of HM of 0.9–2.2/1000 pregnancies in periods from 1975 to 2009^8,22,23,25,32,34,36^.

Ideally, for calculating the incidence of HM, the denominator should represent the population at risk; that is, all conceptuses, both those in pregnancies that ended by live- or stillbirth and those in pregnancies that ended by induced or spontaneous abortion, including very early miscarriages. Therefore, the incidence per recognized pregnancy may be closer to the ideal than the incidence per delivery. However, the number of deliveries can be retrieved more accurately than the number of pregnancies.

We observed a higher average incidence of PHM than of NPHM, although we observed a decrease in the incidence of PHM over time. Others reported a higher incidence of PHM compared to CHM^8,10,11,27,34^. Eysbouts et al. found a clear preponderance of PHMs from 1996 to 2010 in the Netherlands, however from 2010, CHMs were observed more frequently than PHMs^6^. The clinical appearance of PHMs is more subtle than that of CHMs and PHMs often remain undiagnosed sonographically^29,38^. Thus, the probability that a PHM is not being sent for histopathological examination is higher than the probability that a CHM is not being sent. Likewise, the histopathological characteristics of a PHM can be inconspicuous and the distinction between non-molar hydropic miscarriages and PHMs can be very difficult^1^. Likely the true frequency of PHM is higher than the frequency of CHM.

In agreement with several other studies^4,11,25,26^, we found a high incidence of HM in both adolescent women and women at advanced age, supporting the hypothesis that fertilization of an abnormal oocyte is more likely to occur in these age groups^39^. Consistently with other reports^27,39^, we found the association between the incidence of HM and maternal age to be more prominent for NPHM than for PHM, supporting that there is a fundamental biological difference between CHM and PHM.

The frequencies of second and third HMs were 40/1976 (2%) and 1/40 (3%), respectively. In other studies a similar frequency of second HMs was observed, whereas a higher frequency of third HMs was observed^40–42^. The facts that the second mole was of the same type as the first mole in the majority of women, and that in the woman with three HMs all were NPHMs, are also consistent with the findings reported by others^41,42^. While most HMs occur sporadically, some women with recurrent HM (that mostly are CHMs), have a rare autosomal recessive predisposition to molar pregnancies^43,44^. Hence, the frequency of women with repeated HMs, will be higher in populations with a high frequency of consanguinity. In Denmark, the frequency of consanguineous marriages is low. Possibly this explains the low number of women with more than one recurrent HM in our study.

## Conclusion

The incidence of HMs in Denmark may be among the highest in Europe. Unlike the incidence reported from most other European studies, the incidence of registered HM in our study is decreasing, especially for PHM. The decrease may partly be explained by a diminished fraction of miscarriages subjected to pathological examination.

## Supplementary information

Supplementary Tables.
