# Supplementary material for: Decreasing incidence of registered hydatidiform moles in Denmark 1999–2014
Source: Sci Rep. 2020 Oct 12;10:17041. doi: 10.1038/s41598-020-73921-4 (PMC7552396; doi:10.1038/s41598-020-73921-4)
Supplement: Supplementary file 1 — Supplementary Tables. [file 41598_2020_73921_MOESM1_ESM.pdf]

Decreasing incidence of registered hydatidiform moles in Denmark 1999-2014

Helle Lund[1,2], Mogens Vyberg[1,2], Helle Højmark Eriksen[3], Anni Grove[1] , Annette Østergaard Jensen[1], Lone Sunde[4,5]

[1]Department of Pathology, Aalborg University Hospital, Ladegaardsgade 3, 9000 Aalborg, Denmark, [2]Department of Clinical Medicine, Aalborg University, Søndre Skovvej 15, 9000 Aalborg, Denmark, [3]Unit of Epidemiology and Biostatistics, Aalborg University Hospital, Søndre Skovvej 15, 9000 Aalborg, Denmark, [4]Department of Biomedicine, Aarhus University, C. F. Møllers Allé 6, 8000 Aarhus C, Denmark, [5]Department of Clinical Genetics, Aalborg University Hospital, Ladegaardsgade 5, 9000 Aalborg, Denmark

Supplementary material, part A Incidence of hydatidiform mole (HM) in Denmark 2007-2014. Results from the Danish Pathology Registry

Year	Deliveries[a] n	HMs[b] n	Induced[c] abortions n	Miscarriages[c] n	Ectopic pregnancies[c] n	Pregnancies, unknown localisation[c] n	Total n	Incidence of HM	
								per 1000 pregnancies	per 1000 deliveries
2007	63651	109	16888	10173	1014	0	91835	1.19	1.71
2008	64462	143	17730	10363	1002	0	93700	1.53	2.22
2009	62368	107	17732	10122	975	0	91304	1.17	1.72
2010	62956	120	17648	9642	957	0	91323	1.31	1.90
2011	58627	92	17171	9170	986	0	86046	1.07	1.57
2012	57525	101	17036	8867	840	0	84369	1.20	1.76
2013	55418	100	16783	8882	847	30	82060	1.22	1.81
2014	56491	91	16360	8753	987	693	83375	1.09	1.61
Total	481498	863	137348	75972	7608	723	704012	1.23	1.79

[a]Deliveries registered in the Danish Birth Registry. [b]Hydatidiform moles registered in the Danish Pathology Registry. [c]Recorded pregnancies (induced abortions, miscarriages, ectopic pregnancies and pregnancies, unknown localization (introduced 2012) at Danish hospitals and general practitioners registered in the Danish database for Early Pregnancy and Abortion (TiGrAb, annual report 2016). Abbreviations: HM, hydatidiform mole.

Supplementary material, part B Incidence of hydatidiform mole (HM) in Denmark 1999-2014. Results from the Danish Pathology Registry

Year	Without ÆYYY00[a]		With ÆYYY00[b]		NPHM[c] + PHM[d]			Deliveries[e] n	Incidence per 1000 deliveries					
									Without ÆYYY00[a]			All[a+b]		
	NPHM[c] n	PHM[d] n	NPHM[c] n	PHM[d] n	Without ÆYYY00[a] n (%)	With ÆYYY00[b] n (%)	All[a+b] n		NPHM[c]	PHM[d]	NPHM[c] + PHM[d]	NPHM[c]	PHM[d]	NPHM[c] + PHM[d]
1999	48	99	5	15	147 (88.0)	20 (12.0)	167	65524	0.73	1.51	2.24	0.81	1.74	2.55
2000	58	83	11	15	141 (84.4)	26 (15.6)	167	66399	0.87	1.25	2.12	1.04	1.48	2.52
2001	52	65	12	13	117 (82.4)	25 (17.6)	142	64616	0.80	1.01	1.81	0.99	1.21	2.20
2002	48	84	8	10	132 (88.0)	18 (12.0)	150	63235	0.76	1.33	2.09	0.89	1.49	2.37
2003	55	68	5	5	123 (92.5)	10 (7.5)	133	63831	0.86	1.07	1.93	0.94	1.14	2.08
2004	52	53	5	6	105 (90.5)	11 (9.5)	116	63918	0.81	0.83	1.64	0.89	0.92	1.81
2005	48	56	11	11	104 (82.5)	22 (17.5)	126	63595	0.75	0.88	1.63	0.93	1.05	1.98
2006	49	51	3	9	100 (89.3)	12 (10.7)	112	64403	0.76	0.79	1.55	0.81	0.93	1.74
2007	40	43	13	13	83 (76.1)	26 (23.9)	109	63651	0.63	0.68	1.30	0.83	0.88	1.71
2008	68	53	13	9	121 (84.6)	22 (15.4)	143	64462	1.05	0.82	1.87	1.26	0.96	2.22
2009	42	42	12	11	84 (78.5)	23 (21.5)	107	62368	0.67	0.67	1.34	0.87	0.85	1.72
2010	65	41	7	7	106 (88.3)	14 (11.7)	120	62956	1.03	0.65	1.68	1.14	0.76	1.90
2011	35	42	3	12	77 (83.7)	15 (16.3)	92	58627	0.60	0.72	1.32	0.65	0.92	1.57
2012	48	50	2	1	98 (97.0)	3 (3.0)	101	57525	0.83	0.87	1.70	0.87	0.89	1.76
2013	38	48	4	10	86 (86.0)	14 (14.0)	100	55418	0.69	0.87	1.56	0.76	1.05	1.81
2014	33[f]	42	3	13	75 (82.4)	16 (17.6)	91	56491	0.58[f]	0.74	1.32[f]	0.64[f]	0.97	1.61[f]
Total	779[f]	920	117	160	1699 (86.0)	277 (14.0)	1976	997019	0.78[f]	0.92	1.70[f]	0.90[f]	1.08	1.98[f]

[a]HMs registered with a HM code without the moderator code ÆYYY00 indicating "suspicion of". [b]HMs registered with a HM code with the moderator code ÆYYY00 indicating "suspicion of". [c]HMs registered with the code M91000 (hydatidiform mole/complete hydatidiform mole) or M910A0 (hydatidiform mole, not otherwise specified). [d]HMs registered with the code M91030 (partial hydatidiform mole). [e]Deliveries registered in the Danish Birth Registry 1999-2014. [f]Including two HMs registered with the code M910A0 (hydatidiform mole, not otherwise specified). Abbreviations: HM, hydatidiform mole; NPHM, a HM not registered as a PHM; PHM, partial hydatidiform mole.

Supplementary material, part C Incidence of hydatidiform mole (HM) in Denmark 2007-2014 calculated both per 1000 pregnancies and 1000 deliveries

Year	Incidence per 1000 pregnancies						Incidence per 1000 deliveries					
	Without ÆYYY00[a]			With or without ÆYYY00[b]			Without ÆYYY00[a]			With or without ÆYYY00[b]		
	NPHM[c]	PHM[d]	NPHM[c] + PHM[d]	NPHM[c]	PHM[d]	NPHM[c] + PHM[d]	NPHM[c]	PHM[d]	NPHM[c] + PHM[d]	NPHM[c]	PHM[d]	NPHM[c] + PHM[d]
2007	0.44	0.47	0.91	0.58	0.61	1.19	0.63	0.68	1.30	0.83	0.88	1.71
2008	0.73	0.57	1.3	0.86	0.66	1.53	1.05	0.82	1.87	1.26	0.96	2.22
2009	0.46	0.46	0.92	0.59	0.58	1.17	0.67	0.67	1.34	0.87	0.85	1.72
2010	0.71	0.45	1.16	0.79	0.52	1.31	1.03	0.65	1.68	1.14	0.76	1.90
2011	0.41	0.49	0.90	0.44	0.63	1.07	0.60	0.72	1.32	0.65	0.92	1.57
2012	0.57	0.59	1.16	0.59	0.60	1.20	0.83	0.87	1.70	0.87	0.89	1.76
2013	0.46	0.58	1.05	0.51	0.71	1.22	0.69	0.87	1.56	0.76	1.05	1.81
2014	0.40[e]	0.50	0.90[e]	0.43[e]	0.66	1.09[e]	0.58[e]	0.74	1.32[e]	0.64[e]	0.97	1.61[e]
Total	0.52[e]	0.51	1.04[e]	0.61[e]	0.62	1.23[e]	0.77[e]	0.75	1.52[e]	0.88[e]	0.91	1.79[e]

[a]HMs registered with a HM code without the moderator code ÆYYY00 indicating “suspicion of”. [b]HMs registered with a HM code with or without the moderator code ÆYYY00 indicating “suspicion of”. [c]HMs registered with the code M91000 (hydatidiform mole/complete hydatidiform mole) or M910A0 (hydatidiform mole, not otherwise specified).). [d]HMs registered with the code M91030 (partial hydatidiform mole). [e]Including two HMs registered with the code M910A0 (hydatidiform mole, not otherwise specified). Abbreviations: HM, hydatidiform mole; NPHM, a HM not registered as a PHM; PHM, partial hydatidiform mole

.

Supplementary material, part D Time interval between first and second hydatidiform mole registered in the Danish Pathology Registry

Case	Interval[a]	Case	Interval[a]	Case	Interval[a]	Case	Interval[a]
1	181	11	301	21	600	31	1393
2	186	12	308	22	629	32	1490
3	213	13	353	23	718	33	1497
4	224	14	356	24	891	34	1688
5	228	15	384	25	904	35	2339
6	238	16	407	26	910	36	2594
7	253	17	435	27	979	37	2645
8	272	18	459	28	1014	38	3639
9	277	19	497	29	1099	39	4508
10	281	20	564	30	1105	40	5702

[a]Interval in days; median=582 days
